# Feeling the force: Returning haptic signals influence effort inference during motor coordination

**DOI:** 10.1038/srep02648

**Published:** 2013-09-12

**Authors:** G. Ganesh, R. Osu, E. Naito

**Affiliations:** 1 Center for Information and Neural Networks (CiNet), National Institute of Information and Communications Technology, 1-4 Yamadaoka, Osaka University Campus, Suita, Japan 5650871; 2 ATR Computational Neuroscience Laboratories, 2-2-2, Hikaridai, Seika-cho, Soraku-gun, Kyoto, Japan 6190288; 3 Graduate School of Medicine, Osaka University, 1-4 Yamadaoka, Suita, Japan 5650871

## Abstract

Our brain is known to automatically optimize effort expenditure during motor coordination, such that for example, during bimanual braking of a bicycle, a well-oiled brake will automatically be used more than a corroded, heavy brake. But how does our brain infer the effort expenditure? All previous motor coordination models have believed that the effort in a task is known precisely to our brain, solely from the motor commands it generates. Here we show that this belief is incorrect. Through experiments and simulation we exhibit that in addition to the motor commands, the returning haptic signals play a crucial role in the inference of the effort during a force sharing task. Our results thus elucidate a previously unknown sensory-motor association that has major ramifications for our understanding of motor coordination and provides new insights into how sensory modifications due to ergonomics, stroke and disease can affect motor coordination in humans.

The human motor system is highly redundant. A simple task like picking up a glass of water on the table can be performed using numerous arm trajectories with different spatial and temporal characteristics, and using different muscles. Still, it is observed that individuals repeatedly choose a particular set of muscles and trajectories from the available options for any task. In the optimization framework, this regularity is explained as the minimization of a set of scalar variables that our brain regards as critical during a task^1^. While the variables considered by the human central nervous system (CNS) to determine motion are still debated^10^,^11^, the task error and effort expenditure are probably the most well accepted and have been shown to accurately explain many aspects of motor behaviors such as eye saccades, arm movements^1^,^2^,^3^,^4^ and bimanual coordination^5^,^6^. But, to minimize the error and effort, the CNS needs to calculate them online during movements. While task error is believed to be estimated by the CNS through the integration of the sensory predictions with the sensory observations^12^,^13^, the popular belief about effort is that this is known precisely to the CNS^1^,^2^,^3^,^4^,^5^,^8^, solely from the motor commands it generates. The possible involvement of the sensory feedbacks in effort estimation has been neglected because the motor commands are considered to be known to the CNS and sufficient to give an accurate estimate of effort.

However, the one fact that is well accepted about the sensory and motor systems is that they form a closely coupled unit where functions performed by one system are, more often than not, strongly affected by the other^1^,^14^. In this study we examined if this is true also for effort inference, and hypothesized that in addition to the motor commands, the haptic feedback determines effort expenditure in motor tasks involving contact force. We focused purposely on the haptic sensory feedback and contact force production because they characterize a majority of motor tasks and because the role of haptic feedback has been reported to be particularly unclear in previous studies that examined cognitive *feeling of effort*^15^,^16^ in humans. Critically, if true then our hypothesis would exhibit a previously unknown sensory-motor association, providing new insights into how haptic deprivations due to ergonomics, disease and stroke can affect human motor behaviors.

To examine the role of haptic signals in the inference of the effort expenditure during motor coordination, we chose a bimanual two finger force sharing task^5^. While this task gives a target for the total force applied by the two fingers, the subject's CNS is free to choose how the exerted force is distributed between the fingers. It has been previously exhibited^5^ that the force distribution between the fingers in this task is determined by the force variability in each finger and the effort (force) applied by each finger. We checked if and how the force distribution between the two fingers is affected when the tactile sensation in one of the fingers is reduced using a glove without changing its force variability (hence task error).

## Results

### Experiment-1

**Figure 1:**
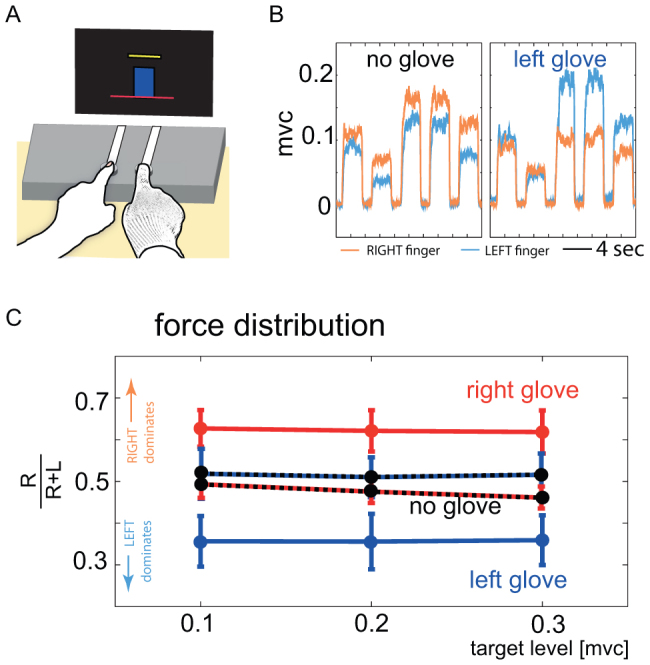
Bimanual two-finger force sharing task. (A) The task required subjects to press on two force sensors isometrically with their index fingers. In one session, they wore an elastic cloth glove (right hand in figure) on one of their hands. The subjects were given a visual feedback of the total force they applied (blue bar) which they aimed to match the given target level (yellow bar). (B) The force production by the left (cyan trace) and right (orange trace) fingers by a representative subject in two sessions without and with a (left) glove. The target levels were calibrated to the maximum voluntary contraction (mvc) of individual subjects. (C) Gloved finger takes more load: The force distribution in the task by 12 subjects across three target force levels (0.1 mvc, 0.2 mvc and 0.3 mvc) was quantified as the ratio of the right finger force to the total force (R/(R+L)) and averaged for the no glove sessions across subjects for the left gloved subjects(black-blue trace) and right gloved subjects (black-red trace), and for the sessions with left (blue trace) and right (red trace) gloves. A force distribution of 0.5 indicates that both fingers apply equal force, a higher value shows that the right finger applies more force than the left while a force distribution value less than 0.5 indicates that the left finger applies more force than the right.

Twelve neurologically healthy, naïve (one left-handed) subjects participated through two experiments in our study. In Experiment-1, they pressed on two force sensors isometrically (Fig. 1A) with their left and right index fingers. They were given a visual feedback of the sum of their finger forces which they matched to one of three pseudo-randomly presented target levels (yellow bar in Fig. 1A). The targets were pre-calibrated to 0.1, 0.2 and 0.3 times of the maximum voluntary contraction (mvc) in each subject. The target presentation time (4 sec) was set such that all subjects could achieve steady state during force production. The subjects performed this two-finger task over two sessions; one when their hands were free (no glove session) and one when they wore an elastic cloth glove in one of the hand (glove session). The subjects were divided into two groups; one wore the right glove in the gloved sessions, while the other wore the left glove in the gloved sessions. The glove decreased the tactile sensation in the finger. The decrease in tactile sensation was confirmed by checking for a decrease in tactile perception in the finger using Von Frey's hairs (see detailed methods) and quantified using Experiment-2. The force profile from a representative subject is shown for with and without the left glove (Fig. 1B).

Fig. 1C shows the steady-state force distributions during the sessions measured as the ratio of the right finger force to the total force (R/(R+L)). This was computed over the last 2 seconds of each target trial across subjects so as to ensure the steady state was achieved. The force distribution in the no glove session (black trace) varied between subjects, but overall the subjects utilized the two fingers equally in this session in agreement with previously reported observations^5^. However, with a 2 way ANOVA between the 3 sessions (2 glove and one no-glove) and 3 target levels, we observed that on using a glove, the subjects consistently utilized their gloved finger more (p < 10^−5^) in the task irrespective of the target level (p = 0.94), with no interactions (p = 0.99) between the glove and target level. If they wore a glove in their left hand, they used the left finger more (T(17) = 7.76, p < 10^−6^, one sample T-test across the target levels and subjects) and the converse if they wore a glove on their right hand (T(17) = 6.16, p < 10^−4^, one sample T-test across the target levels and subjects). Importantly, the increased use of the gloved finger was observed in every subject and at every target force level. The difference in force distribution (R/(R+L)) across the experiment between the gloved and non-gloved sessions was used as the quantitative value for the change in force distribution (ΔD of Fig. 3,4B) due to the glove in Experiment-1.

**Figure 2:**
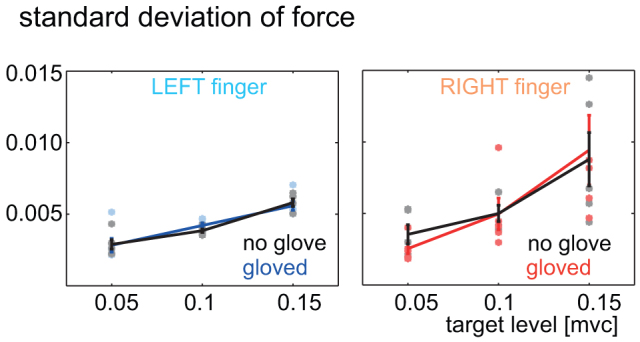
Results from single-finger session. The standard deviation of the force at each target force level shows no significant difference between the gloved (blue/red) and no glove (black) sessions across subjects with the left and right hands (two panels). The individual averages are shown as dots while the across subject average and standard error are represented by solid traces.

While the steady state force applied by a finger was affected by the presence of the glove, the standard deviation of the force produced by an individual finger was not. In separate single-finger sessions, the same subjects performed the task with either one of their fingers with and without the glove. Correspondingly the force feedback was also provided only from the one finger while the target levels in these sessions were set to half of the levels presented during the two-finger sessions described above. The standard deviation in the steady state force recorded in no-glove single-finger sessions was compared to that recorded in the gloved single-finger session, using a 2 way ANOVA across the 2 sessions and three target levels from the subjects. The standard deviation of the finger force increased with the level of force (p < 10^−5^ Fig. 2) consistent to the belief that motor signals are associated with a signal dependent noise^7^, but the force variations were same between the gloved and non-gloved sessions for both the left finger (p = 0.89) and the right finger (p = 0.63). This result clearly exhibited that the glove does not affect the variability in the finger force during the task.

### Experiment-2

**Figure 3:**
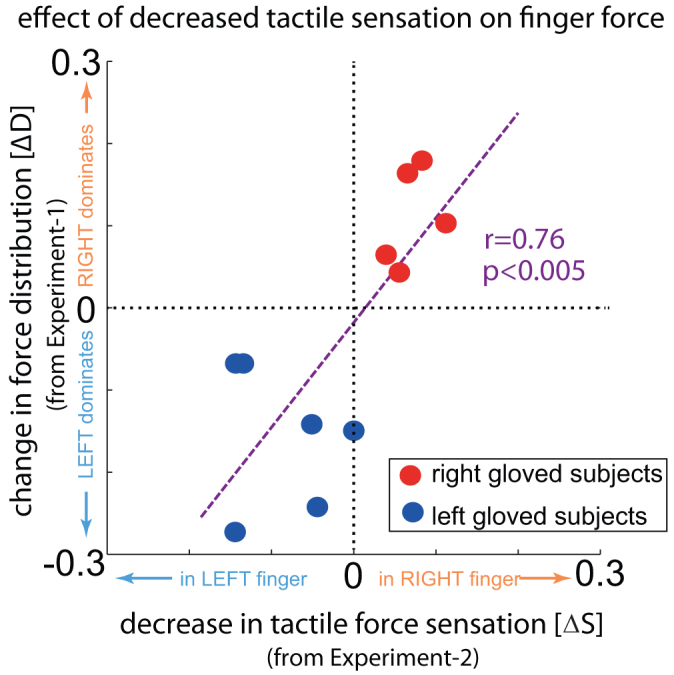
Effect of tactile sensation on force distribution. (A) The average change in force distribution (ΔD from Experiment-1) in every individual was plotted against the individual's average change in force sensation (ΔS from Experiment-2). A positive ΔD indicates an increase in the right finger force while a negative ΔD indicates an increase in the left finger force. Similarly a positive ΔS indicates a decrease in tactile sensitivity in the right finger while a negative ΔS indicates a decrease in tactile sensation in the left finger. A significant linear correlation (p < 0.005) was observed between the decrease in force sensation (ΔS) and the change in force distribution (ΔD) across subjects.

**Figure 4:**
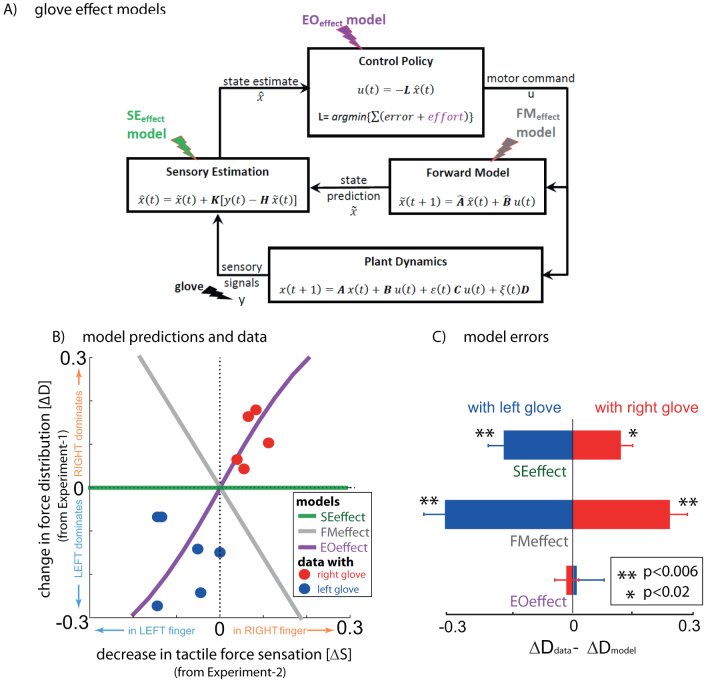
Model predictions and data. (A) A general representation of the optimal feedback control framework^30^ with the three glove effect models. The glove effects the tactile sensation (y). The SEeffect (green) assumes the effect of the glove to be restricted to the sensory signal (y). The FMeffect model (grey) assumes the glove affects the forward model as well by inducing an error in the estimated task input matrix (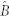). The EOeffect model (violet) assumes the glove affects, not just the sensory signal but also the effort optimization during the task. (B) The models made distinct predictions on how a change in tactile sensation (ΔS) as measure in Experiment-2 would change the force distribution (ΔD) in Experiment-1. (C) The difference between the change in finger force distribution in the data (ΔD_data_) and the finger force distribution predicted by the model (ΔD_model_) was significantly different for the SEeffect and FMeffect models during both left (blue bars) and right (red bars) glove sessions. On the other hand, the data conformed well to the EOeffect model for both the left glove (p = 0.79) and right glove (p = 0.47) subjects.

Next, in Experiment-2, we quantified the change in tactile sensation induced by the glove using a force perception task. All the subjects from Experiment-1 participated in Experiment-2, which also required two finger force production but with a different protocol. The subjects were first instructed to match a given force target (which was same as the single-finger sessions) with only their non-gloved finger and aided by the visual feedback of the non-gloved finger force. While maintaining the force with the non-gloved finger, they were then asked to apply a force with the gloved finger to ‘match the haptic feeling’ in the non-gloved finger. No visual feedback was provided of the force applied by the gloved finger. Separately, the subjects also performed a control session in which they performed the exact same procedure without a glove in either hand; i.e. the subjects first matched the force target with a finger that was previously ungloved and then matched the haptic feeling with the finger that was previously gloved. The difference in force distribution (R/(R+L)) between these two sessions was used as the quantitative value for the change of force sensation (ΔS in Fig. 3,4) due to the glove. Overall, the force sensation was observed to decrease by 11.75 ± 4.0 SE% in the gloved finger across subjects.

Experiment-1 and Experiment-2 together demonstrated that a decrease in tactile sensation (ΔS) in a finger led to a change in force distribution (ΔD) in our force sharing task. Indeed, we observed a significant positive correlation (Fig. 3; Pearson's R = 0.761, p < 0.005, one subject was omitted as a outlier) between ΔS and ΔD across subjects exhibiting that a decrease in tactile sensation due to the glove was accompanied by an monotonic increase in the force assigned to the gloved finger. However, our experiments did not clarify how and why the glove leads to a change in force distribution. To concretely exhibit that the observed effect was due to tactile signals being utilized for effort inference, we modeled the subject behavior in our two experiments.

While a static optimization of a cost function of error and effort (finger force) would have been sufficient for this purpose, we purposely choose a dynamic optimization model to clearly show that the behavior we get cannot be explained by the changes in the forward model or in the force transients due to the glove. The optimal feedback control^2^ (Fig. 4A) architecture provided us with an elegant way to clarify these points.

### Modeling and simulations

We defined the muscle dynamics in our model (**A**, **B** in Fig. 4A) as in previous studies^8^,^9^ and considered signal dependent (scaling matrix **C**) and signal independent (scaling matrix **D**) noise. Matrices **Q**, and **R** defined the control policy in the two experiments. Matrix **H** defined the noisy sensory observations available to the subjects, which they combine with the sensory predictions available from the forward model to achieve a current sensory estimate. We considered both the visual feedback of finger force (sum of the finger forces in Experiment-1 and of the non-gloved finger in Experiment-2) provided during the task, and the tactile sensation available to the subjects from each finger (see methods). *ε* and ξ were Gaussian random in time with mean 0 and variance 1.

Next we modeled the effect of the glove. The glove obviously effects the tactile sensory feedback (y in Fig. 4A), but the glove cannot change the relation between the applied motor commands and the generated force; i.e the state matrix (**A**) and the input matrix (**B**) are not expected to be affected by the glove. This is because our task was isometric with no change in body posture between the gloved and the non-gloved sessions. Furthermore, no differences were noticed in the force variance due to the glove (Fig. 2) and hence the noise scaling (**C, D**) was also unaffected by the glove. We therefore considered the three remaining possibilities by which the glove could have affected the behavior in our experiments.

First, in the Sensory-estimation-effect (SEeffect) model we considered the possibility that the glove affects only the sensory estimation process. This is the simplest effect model that assumes that the glove effect is restricted to the change in the tactile sensory feedback (y in Fig. 4A) and does not affect any other element of the control process. However, a change in tactile sensory signals does not affect the task error feedback, which was visual in our task. On the other hand, the effort was determined by the motor commands in the SEmodel. Consequently, the SEeffect model predicts the steady state force sharing to remain unchanged (green line, Fig. 4B) due to a change in tactile sensation. This prediction was obviously different from our data (p < 0.02, Fig. 4C) and exhibited that the glove affects more than just the sensory feedback in our task.

Second, in the Forward-model-effect (FMeffect) model we considered the possibility that, in addition to the effect on the tactile feedback as in the SEmodel, the glove also affects the forward model of the task (see Fig. 4A). In other words, the reduction in tactile signal due to a glove is misinterpreted by the CNS as a loss of efficiency, where a same effort produces less force output by the gloved finger compared to the non-gloved finger. This can be represented by a change in 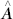 or/and 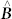 in Fig. 4A. Consequently, as motor coordination is determined by effort minimization, the FMeffect model predicts that with the decrease in tactile sensation in the gloved finger the CNS would assign progressively more load to the non-gloved ‘more efficient’ finger in the force sharing task (grey line, Fig. 4B). However, this prediction is converse to the force distribution change we observed in our experiments (compare grey line and data points in Fig. 4B, p < 0.006 in Fig. 4C). Therefore, we ruled out the possibility that the force distribution changes observed in our task were due to a change in the forward model due to the glove.

Finally, in the Effort-optimization-effect (EOeffect) model we assumed that in addition to the effect on the tactile feedback, the glove affects the control policy, specifically the effort optimization in our task. This model conforms to our hypothesis and assumes that the effort expenditure during the task is determined not only by the motor commands (u) but also by the tactile feedback. This possibility can be represented by a change in cost function in Fig. 4A (see methods) and predicts that the force applied by a finger to increase as the tactile sensation decreases (violet trace in Fig. 4B). The experimental observations agreed extremely well with the EOeffect model (left glove: T(5) = 0.28, p = 0.79; right glove: T(4) = 0.78, p = 0.47, see Fig. 4C). The simulations thus clearly show that the force distribution observed in our task (Fig. 2C, Fig. 3) can only be explained if the tactile signals are utilized in the inference of effort expenditure during our coordination task. A decrease in tactile feedback in a finger is mis-interpreted by the CNS as reduced effort by the finger during force sharing, and is compensated with an increase of the force assigned to the finger.

## Discussion

Understanding motor coordination-how the brain distributes and controls a motor task across the available choice of muscles and joints, is fundamental for our understanding of human motor behavior in health and pathology and thus has been a key research goal in motor neuroscience^1^. It is believed that motor coordination in humans is achieved utilizing sensory feedbacks and optimizing the error and effort expenditure during a task. However, up till now, the role played by sensory feedback was believed to be restricted to the inference of task error^12^,^13^ while effort expenditure was believed to be known solely from the motor commands^15^. In this study we showed that, in addition to the motor commands, the haptic feedback plays a critical role in determining the effort cost our CNS infers during a motor task. With our experiments, we first exhibited that a reduction in the tactile sensation in a finger affects the force distribution in a force sharing task, with a monotonic increase in the force applied by the affected finger. Next, we showed that the change in force distribution cannot be explained by a change in task variability due to the glove (Fig. 2), a tactile bias alone (SEeffect model) or an error in the forward model (FMeffect model) due to the glove. The force distribution change in our task occurred because the force production and hence the effort expenditure was inferred from the tactile feedback received during the task (EOeffect model, Fig. 4).

Note that it is possible to explain our finding by constructing other cost functions^17^, for example, one that includes not just error and effort but also a sensory term that equalizes the sensory feeling between the fingers. However, in order to construct the right cost function, we need to first know the cost variables considered by the CNS; a knowledge that is still debated^10^,^11^ and out of the scope of this study. Therefore at present we choose to explain our results in terms of the cost function (including error and effort) that has been most successful in the past to explain motor behaviors^1^,^2^,^3^,^4^,^5^,^6^ including that in the finger force sharing task that we use here^5^. Future studies that isolate definite cost variables used by the human may explain our results differently. However, in the least, our results clearly exhibit that sensory feedbacks determine more than just the task error during motor action and this result has several important implications.

Primarily, it encourages a rethink of our understanding of ‘effort optimization’ and its modeling. In this study we specifically investigated tactile feedback; a feedback that is present only in the presence of external forces and during contact with another object. But our elucidation of the sensory role in effort inference suggests similar roles may be played by proprioception in motor tasks that do not involve physical contact, such as reaching. Our results support the theory that our CNS may interpret sensory signals as covertly generated motor commands^14^ and elucidate that, similar to task error, effort expenditure values may also be noisy and delayed and require forward models^18^ for their estimation.

Second, our results can provide clues to explain several seemingly unusual results in previous motor studies. Grip studies have regularly exhibited that humans are able to remember and produce predictive grip forces while handling commonly gripped objects^19^. However, patient grip studies and studies in ergonomics have observed that the grip forces for even well-known objects increase when the somatosensory feedback is either absent^20^ or attenuated by a glove over the hand^21^,^22^. These observations are well explained if the returning tactile feedback is considered to determine the generation and control of predictive finger forces. Motor studies have recently highlighted the presence of hysteresis in motor behaviors^23^,^24^,^25^,^26^ where certain past behaviors are maintained even though they are not optimal with respect to the effort cost. However, it has been unclear which component of the sensory-motor system is responsible for this hysteresis. Our finding shows that the hysteresis might be computationally explained as a persisting bias in the control policy resulting from sensory factors that are known to suffer from *after-effects* and illusions^27^.

Finally, our result provides new perspectives for our understanding of neural abnormalities and their rehabilitation. Our observation suggests alternate computational perspectives to explain behaviors observed in Micrographia and Parkinson Disease, which were previously associated with mis-calculations in the effort expenditure^28^,^29^. These may be alternatively explained by a deficiency in the sensory-motor transformation circuits. The new associations between motor action and sensory feedback observed in our study elucidate how haptic deteriorations can affect motor control in stroke and accident rehabilitations. The finding that a decrease in tactile sensation leads to the increased use of a limb has obvious benefits for rehabilitation. While it supports recent results exhibiting the effectiveness of sensory rehabilitation in restoring motor function^31^, it also encourages novel rehabilitation paradigms where a purposely induced reduction of haptic input may help augment voluntary motor output.

## Methods

### Experiment

12 neurologically healthy, naïve subjects (age between 23–45; one female, one left handed male). Their handedness was verified using the Oldfield handedness inventory. The experiment was approved by the local ethics committee at ATR and subjects provided informed consent for their participation. The subjects participated in one force calibration session, five force production sessions, 2 passive force perception session (data not used in this study) and 2 active force perception sessions. The force production sessions were classified as Experiment-1 while the force perception sessions were classified as Experiment-2.

### Session types

#### Force calibration session

The subjects sat comfortably on a chair in front of a table on which they were required to press two force sensors with the index fingers of their left and right hands while the other fingers were closed into a fist (see Fig. 1A). The chair height was adjusted as per the subjects comfort. Visual feedbacks were provided on a computer screen placed on the table in front of them.

In the calibration session the subjects were asked to press the force sensor as strongly as possible four times −2 seconds each time followed by a 2 second rest. The presses were cued by a visual feedback on the computer monitor. The average of the force values over the last second in each of the four presses were used to define the maximum voluntary contraction (mvc) for the left and right fingers separately. These mvc values were used to normalize the force values presented as feedback to the subjects in the next sessions. The force distribution was quantified as the ratio of the right finger force and the total force (R/(R+L)) and plotted for target across subjects in Fig. 1C. The difference in force distribution (R/(R+L)) across the experiment between the gloved and non-gloved sessions was used as the quantitative value for the change in force distribution (ΔD of Fig. 3,4B) in Experiment-1. Therefore, a positive ΔD indicates an increase in the right finger force while a negative ΔD indicates an increase in the left finger force.

#### Force production session (Experiment-1)

In the five force production sessions, the subjects pressed either one force sensor with one finger (single finger session) or both force sensors with both index fingers (two finger sessions). Each subject also performed the two kinds of sessions wearing an elastic cloth glove on either their left or right hand. The five force production sessions in our Experiment-1 were −1) single finger press with right hand finger, 2) single finger press with left hand finger, 3) two finger press with no glove, 4) two finger press with glove on one hand and 5) single finger press with the gloved hand. Note that the order of the sessions was randomized across subjects.

During the five sessions the subjects were provided with a visual feedback of the applied finger force represented by a blue bar on a computer screen. The feedback corresponded to the sum of their finger forces in case of the two fingers sessions and corresponded to the single finger forces in the single finger sessions. The task required the subjects to match the blue bar to the provided yellow targets on the screen. The targets were set at 0.1, 0.2 and 0.3 mvc in the two fingers sessions and as 0.5, 0.1, and 0.15 mvc in the single finger sessions. Each target was presented 6 times in a session. The 18 targets (3 levels × 6 times) were presented in a pseudo random order where each target was presented for 3 seconds followed by a rest of 1 second.

#### Passive force perception (Experiment-2) (note that this data is not used in this study)

zThe subjects performed two passive force perception sessions-without a glove, and with a glove on one hand. In each session, they were asked to close their eyes and place their two hands on their lap, palm facing up. The experimenter then pressed a Von Frey hair^32^ (chosen pseudo-randomly from 0.02 g, 0.04 g, 6 g, 15 g, 180 g and 300 g) on the non-gloved finger, followed by pressing another hair on the gloved finger. In each trial a subject was asked to consider the load feeling of the press on the non-gloved hand to be level 5 on a scale of 1 and 10 and asked to rate the subsequent press on the gloved hand relative to the first press. This process was repeated 25 times in one session. A similar procedure was repeated for the no-glove session where the order in which the two fingers were pressed was kept the same in the session without gloves. Of all the presses in one session only 15 pseudo randomly distributed trials, when the same Von Frey Hair was pressed on both fingers, were used in the passive tactile sensation analysis. The % difference in these 15 scores between the gloved and non-gloved sessions were defined as the % change in passive tactile sensation. Every subject reported a decrease in passive tactile sensation. Across subjects, the passive tactile sensation decreased by 19.82%.

#### Active force perception (Experiment-2)

The subjects pressed force sensors with both fingers in two sessions- one with no gloves and with a glove in one hand. However, *visual feedback was provided only for the force applied by the non-gloved finger*. The targets were similar to the single finger sessions in Experiment-1. In the gloved session (when one hand was gloved) the subject were asked to first match the non-gloved finger force to the target, maintain this level following which they were asked to press the gloved finger to ‘match the tactile feeling’ in the non-gloved finger. The same order of finger press was also maintained in the non-gloved session. The behavior was again classified by the ratio of the right finger force and the total force (R/(R+L)) similar to Experiment-1. A difference in force distribution between the gloved and no gloved sessions is plotted as the abscissa of Fig. 3, 4B as a measure of decrease in tactile sensitivity (ΔS). Therefore, a positive ΔS indicates a decrease in tactile sensation in the right finger (as then the right finger would be pressed more to equate to the left force) while a negative ΔS indicates a decrease in tactile sensation in the left finger.

#### Session order

Order of presentation of the five force production sessions and two passive force perceptions sessions were randomized across subjects, while the two active force perception sessions were always performed at the end. In total the two experiments took about 40 minutes to complete.

### Modeling and simulations

#### Task

The task dynamics in our experiment are represented in a discrete time formulation as 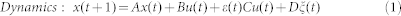

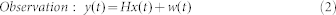

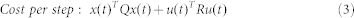
Matrices **A**, **B** in equation (1) were defined assuming that the control signal *u(t)* is transformed into the finger force *f(t)* through a coupled first order filter pair given by 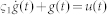, 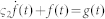 with 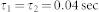
8,9. 
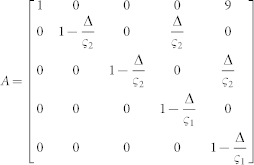
and 
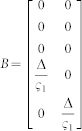
Δ is the simulation frequency which was 0.01 in our simulation. We assume a delay of one time step in the observation. However, similar results can be achieved with larger delays. This is because the delays affect only the transient but not the steady state values of the forces. The state vector is taken as 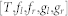, where *T* represents the target force in the task, and *f_l_* and *f_r_* represent the force applied by the left and right fingers. **C** and **D** represent the scaling matrix for the signal dependent noise and random noise while *ε* and ξ are Gaussian random in time with mean 0 and variance 1. 
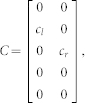
where c1 and c2 were set as 0.01 in our simulation.

The matrices **H**, **Q**, and **R** (Eq. 2 and 3) which define the observation and cost differed between Experiment-1 and Experiment-2 (see following sections).

The state estimation is done through 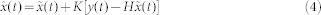
Where 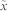 represents the forward model estimation of the state, y represents the online observation while the control policy is represented by 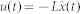. The Kalman gain **K** and optimal control gain **L** are calculated recursively as in^8^ to minimize the expected summation of cost in equation (3).

#### Modeling Experiment-1 and Experiment-2 without the glove

In Experiment-1 the subjects have access to four individual sensory values given by 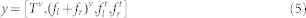
Where the first two terms are given as visual feedback to the subjects. In addition, we assume that the two individual finger forces can also be estimated from the corresponding tactile feedback a *f_l_^t^* and *f_r_^t^*. Hence the observation matrix H in Eq. 2 is given by- 
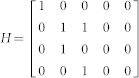


As the task required the subjects to match their summed finger forces to the target (T), the cost becomes 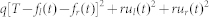, hence 
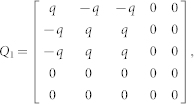
and 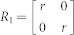
*q* was set to 100 and *r* to 1.

In Experiment-2, when the right hand is gloved, and visual feedback is available only for the left non-gloved hand, the sensory observation is given by


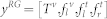
 such that 
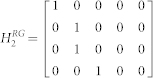


The task required to match the ungloved (left hand) finger force to the target AND match the sensory feeling between the fingers. Therefore the cost becomes 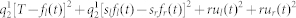 where *s_l_*, *s_r_* represent the linear scaling factor that transform the finger force to the corresponding tactile sensory signals. We take 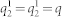 to get: 
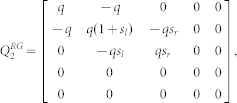
while 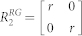


We take q = 100, r = 0.1. Similarly, when the left hand is gloved in Experiment-2, matrices **H** and **Q** change as follows: 
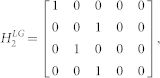

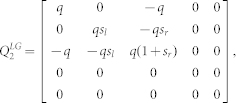
while 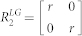


#### Model simulations with the glove

Next, we considered three different effect models by which the glove can affect the above behaviors.

*In all the models*, the glove led to a reduction in tactile sensation in the gloved finger. The tactile feedback of the gloved hand in matrix y (equation 4) was reduced by multiplying the sensitivity (s_l_) to *f_l_^t^*. (see equation 5) to simulate the left glove and (s_r_) to *f_r_^t^*. to simulate the right glove, where 0 < s_l_, s_r_ < 1. The sensitivity in the non-gloved case is considered to be equal to unity.

1. **SEeffect model**—The SEeffect model assumes the glove effect is restricted to the reduction in tactile sensation. To simulate the SEmodel, we first calculated the optimal estimation gains and control signals with the matrices corresponding to the non-gloved sessions, and then to simulate the gloved session, the online tactile observation of the gloved hand in matrix y (Equation 4) was reduced by multiplying the sensitivity (s_l_ or s_r_) in two experiments.
2. **FMeffect model**—The FMeffect model assumes that in addition to the effect on the tactile sensation, the glove also affects the forward model during the task. That is, a change in sensitivity is perceived as a loss of efficiency in the finger-- the same motor command leads to a decreased finger force (though this is false) in the gloved finger. We assume the effect to be limited to the estimated 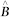 matrix (Fig. 4) utilized during the calculation of the optimal estimator gains and control signals, though qualitatively similar results may be achieved with changes in 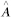. We utilize the same variables −0 < s_l_ < 1 and 0 < s_r_ < 1 to also indicate the forward model changes and simulate the FM model by changing the 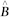 matrix in two experiments as- 
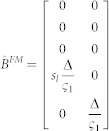
with the left hand glove and 
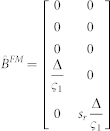
 with the right hand glove.
3. **EOeffect model**—The EOeffect model assumes that the effort cost in a task is determined by not only the motor commands *u* but also by the tactile signals *τ* such that the expected cost per step (equation 3) is infact- 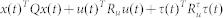

Like before, assuming the tactile signals are linearly related to finger force as 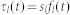 and 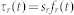 for the left and right hands, the above equation can be rewritten as- 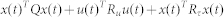


Where R_τ_ is given by 
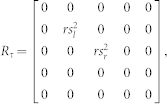
and r = 1.

The EOeffect model can thus be represented by adding **R_τ_** to the **Q** matix in Experiment-1 and Experiment-2. We assume that the glove changes the sensitivities: 0 < *s_l_* < 1.0 with the left glove, and 0 < *s_r_* < 1.0 with the right glove.

Note that s_l_ and s_r_ represent different transformations in the three models, though their value range is same. In our simulations we varied the values of s_l_ and s_r_ between 0 and 0.3 in the two experiments for each model to generate the simulation plots of Fig. 4.

## Author Contributions

G.G. designed the experiment and collected data and made the figures. G.G., E.N. and R.O. developed the study. G.G. and E.N. wrote the main manuscript. All authors reviewed the manuscript.
